# Emerging concepts in the treatment of optic neuritis: mesenchymal stem cell-derived extracellular vesicles

**DOI:** 10.1186/s13287-021-02645-7

**Published:** 2021-12-04

**Authors:** Anagha Aneesh, Alice Liu, Heather E. Moss, Douglas Feinstein, Sriram Ravindran, Biji Mathew, Steven Roth

**Affiliations:** 1 Department of Anesthesiology, College of Medicine, University of Illinois, 835 South Wolcott Avenue, Room E714, Chicago, IL 60612 USA; 2 Departments of Ophthalmology and Neurology & Neurological Sciences, Stanford University, Palo Alto, CA USA; 3 Department of Oral Biology, College of Dentistry, University of Illinois at Chicago, Chicago, IL USA

**Keywords:** Anti-myelin oligodendrocyte glycoprotein associated disease, Autoimmune disorder, Exosome, Extracellular vesicles, MicroRNA, Multiple Sclerosis, Neurodegeneration, Neuromyelitis optica spectrum disorder, Optic neuritis, Optic nerve, Retina, Retinal ganglion cells, Stem cells

## Abstract

**Background:**

Optic neuritis (ON) is frequently encountered in multiple sclerosis, neuromyelitis optica spectrum disorder, anti-myelin oligodendrocyte glycoprotein associated disease, and other systemic autoimmune disorders. The hallmarks are an abnormal optic nerve and inflammatory demyelination; episodes of optic neuritis tend to be recurrent, and particularly for neuromyelitis optica spectrum disorder, may result in permanent vision loss.

**Main Body:**

Mesenchymal stem cell (MSC) therapy is a promising approach that results in remyelination, neuroprotection of axons, and has demonstrated success in clinical studies in other neuro-degenerative diseases and in animal models of ON. However, cell transplantation has significant disadvantages and complications. Cell-free approaches utilizing extracellular vesicles (EVs) produced by MSCs exhibit anti-inflammatory and neuroprotective effects in multiple animal models of neuro-degenerative diseases and in rodent models of multiple sclerosis (MS). EVs have potential to be an effective cell-free therapy in optic neuritis because of their anti-inflammatory and remyelination stimulating properties, ability to cross the blood brain barrier, and ability to be safely administered without immunosuppression.

**Conclusion:**

We review the potential application of MSC EVs as an emerging treatment strategy for optic neuritis by reviewing studies in multiple sclerosis and related disorders, and in neurodegeneration, and discuss the challenges and potential rewards of clinical translation of EVs including cell targeting, carrying of therapeutic microRNAs, and prolonging delivery for treatment of optic neuritis.

**Graphical Abstract:**

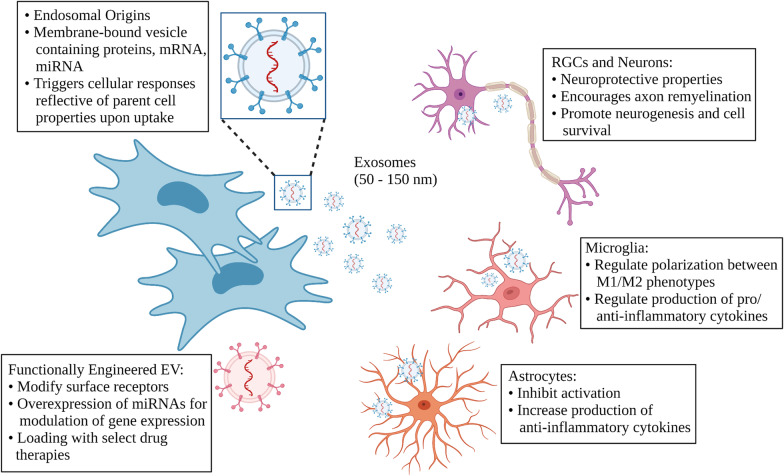

## Background

### Clinical scope of optic neuritis

Optic neuritis (ON) is an inflammatory condition of the optic nerve often associated with central nervous system demyelinating disorders including multiple sclerosis (MS), neuromyelitis optica spectrum disorder (NMOSD), anti-myelin oligodendrocyte glycoprotein associated disease (MOGAD), as well as other systemic autoimmune disorders [1]. ON is characterized by inflammation and demyelination of the optic nerve and inner retinal damage [2].

The presence of antibodies targeting the water channel aquaporin 4 in patients with NMOSD and myelin oligodendrocyte glycoprotein in MOGAD are distinguishing features from MS [3]. The incidence of ON has been estimated in large population studies. In a nationwide database of 44 million people in South Korea, the incidence was 1.04 per 100,000 children, and 3.29 per 100,000 from 2010 to 2016 [4]. In the adult population, it was 5.36 per 100,000 in a hospital discharge database in Spain from 2008 to 2012 [5], and 3.7 per 100,000 in the United Kingdom Health Improvement Network from January 1, 1995, to September 1, 2019. Patients with ON had a significantly higher rate of incident MS (hazard ratio, 284.97; 95% confidence intervals, 167.85–483.81) [6].

Optic neuritis is a clinical diagnosis based on vision loss, optic nerve dysfunction, and absence of symptoms suggesting alternative forms of optic neuropathy. Magnetic resonance imaging typically shows enhancement of the impacted optic nerve segment, and visual evoked potentials demonstrate prolonged latency [7]. In up to 25% of MS patients, ON is the initial inflammatory event [8–10]. Approximately 50% experience ON during the course of their disease [9].

Symptoms of eye pain and blurred vision may worsen over the first few days to 2 weeks, and then gradually improve. Some recover within a month, but recovery could take up to a year. In a 6-month followup of 278 patients in Optic Neuritis Treatment Trial [11] with baseline visual acuity of > 20/50, all improved at least one line of visual acuity, and all except six improved at least three lines [12]. However, the quality of vision, including color or depth perception and contrast sensitivity, may be reduced due to optic nerve demyelination [13, 14]. Vision loss from ON in neuromyelitis optica spectrum disorder is usually more severe than MS-associated ON, with larger scotomas, and bilateral vision loss is common. The risk of long term loss of vision is significantly greater than in MS, with approximately 20–30% functionally blind in the affected eye [15, 16]. Anti-myelin oligodendrocyte glycoprotein associated disease is also associated with significant bilateral visual impairment, but marked ON-related papillitis and long term loss of vision occurs less frequently than in NMOSD [17].

### Pathophysiology of ON

Hallmarks of ON pathology in the acute phase are optic nerve abnormalities and inflammatory demyelination. Predominant T-, B-, and glial cell activation within the nerve increases pro-inflammatory cytokines [18]. Inflammation caused by activated microglia, monocyte-derived macrophages, and CD4- and CD8 + T cells results in reactive gliosis, demyelination, and axonal death [19]. Pro-inflammatory cytokines and cytotoxic factors target myelin-producing oligodendrocytes (OLGs), and oligodendrocyte precursor cells (OPCs), causing apoptosis [20–22], exacerbating axonal demyelination [23–28]. Mature OLGs that survive demyelination are unable to produce new myelin sheaths. Remyelination therefore requires migration and regeneration of oligodendrocytes from OPCs [29, 30]. These acute inflammatory lesions of the afferent visual pathway cause retrograde degeneration of retinal ganglion cells (RGCs) [31, 32]. Retinal nerve fiber layer (RNFL), and inner retinal layer thinning [33, 34] are quantified by optical coherence tomography (OCT). The extent of retinal thinning predicts the disease outcomes [35].

Jin et al. studied the time course of glial activation and its correlation to axon loss in the optic nerve and found that RGC loss was associated with reduced post-synaptic proteins and neurite projections, persistent microglia, and astroglia activation in the inner retina with high levels of iNOS (inducible nitric oxide synthase), IL (interleukin)-1α, TNF (tumor necrosis factor)-α, and C1q (complement component 1q) [23]. Hence, development of therapeutic agents should focus on anti-inflammatory, anti-apoptotic, and remyelinating properties to achieve neuroprotection and neuro-regeneration in the optic nerve and retina.

## Main text

### Available and current treatment

1. Steroids

High dose intravenous steroids are frequently used for treatment of MS-associated ON, based upon results of the now 30-year old Optic Neuritis Treatment Trial [11]. However, there did not appear to be a long-term benefit from a single course of intravenous steroid treatment, and those treated with oral steroids had worse outcomes *vs* intravenous steroids or no treatment at all.

2. Chemotherapy and autologous hematopoietic stem cell transplant

A major focus of research in MS and related neurological disorders is immunosuppression using chemotherapy and immune-depleting antibodies, followed by autologous hematopoietic stem cell transplant (aHSCT) [36]. The rationale is that MS is driven by inflammatory T cells that enter and infiltrate the central nervous system (CNS) and then recirculate in the cerebrospinal fluid (CSF) [37]. Harris et al. investigated T cell clones in the intrathecal compartment in active relapsing remitting MS and found that aHSCT removed most of them, establishing a new immune repertoire in both intrathecal and peripheral blood compartments. However, there have been significant complications that limit this therapy [38].

3. Immune reconstitution therapy

Up to 80% of NMOSD patients have circulating autoantibodies targeting aquaporin-4 (AQP4-IgG) on astrocytes [39]. Monoclonal antibodies that target complement (eculizumab), CD19 (inebilizumab), and interleukin-6 receptor (satralizumab) have been shown effective to prevent attacks in NMOSD in Phase III trials [40], and are a major focus of research and ongoing clinical studies.

The rationale behind anti-complement therapy is the activation of complement in subjects with NMOSD and MOGAD [41, 42]. For eculizumab, the largest study was of 143 patients, showing a significant decrease in rate of relapse [43]. IL­6 is a proinflammatory cytokine increased in NMSOD, and the target of satralizumab. In Phase II studies, satralizumab decreased the rate of relapse [44]. Inebilizumab depletes circulating B cells, and is undergoing Phase II/III trials [45]. The outcomes of major trials of these agents have been extensively reviewed elsewhere [40].

### Limitations and gaps in existing therapies

Steroids are limited by systemic effects, and oral steroids increased the risk of recurrence of ON [11]. In hematopoietic stem cell transplant, a primary concern is secondary autoimmune disease (2ndAD), typically autoimmune cytopenia, idiopathic thrombocytopenic purpura, autoimmune hemolytic anemia, hypo- or hyperthyroidism [46], and myasthenia gravis. 2ndADs were attributed to allogenic imbalances contextualized by graft versus host disease, viral infections, and chronic immunosuppression [46]. Alping et al. compared the monoclonal antibody alemtuzumab, aHSCT, and a reference group of non-induction therapies between 2008 and 2017 [47]. The mortality rate of aHSCT was comparable to the reference group and was lower than that in previous reports, but there was increased infection and thyroid disease *vs* the reference group. A potential limitation of the study includes the lack of a longitudinal component as outcomes were only measured up to 3 years after treatment [48].

Monoclonal antibodies have been approved by the FDA and are safe. The main risk of eculizumab is infection by Neisseria meningitidis, with vaccination now required to prevent meningitis. There are practical considerations with all of the agents, including the need for tightly scheduled and frequent infusions, as well as cost. The estimated yearly cost of eculizumab in the USA is $710,000. The cost of the other agents is about ½ or less that of eculizumab [49].

Due to the recurrent nature of the illness, the lack of long term benefit of steroids, changing nature of the disease with increasing recognition of NMSOD and MOGAD, and occurrence of residual damage to the optic nerve, therapeutic alternatives are needed for ON [18]. In particular, there is a need for agents that can assist with regeneration, and restoring neurological and visual function. Accordingly, in this review we will focus on mesenchymal stem cells and their secreted EVs (exosomes), which have the advantages of autologous treatment, fewer side effects, potential for regeneration of lost neurons, and preservation of vision, and projected lower cost than existing therapies.

### Mesenchymal stem cells

1. General considerations in MS and related disorders:

To promote neuroprotection in ON, attenuation of the inflammatory response, and neuroprotection of glial cells (OLGs, OPCs, astrocytes) and RGCs are key. MSC therapy is a promising approach to reducing the severity of MS-ON by triggering remyelination [50]. MSCs also suppress activation of T-, B-, dendritic, and NK (natural killer) cells [51–54]. It is important to note, however, that most of these studies have been performed in mouse EAE and are not necessarily extrapolatable to other models. Their immunomodulatory properties have been demonstrated in animal models of MS, with intravenous administration of MSCs in EAE mice inhibiting T-cell responses and improving clinical scores [55]. Subsequent studies further demonstrated that MSCs decrease production of inflammatory cytokines, reduce axonal loss, attenuate demyelination, and improve functional recovery in EAE mice and rats when administered prior to disease onset [56–58]. However, all studies in EAE found that while MSCs were able to migrate into the central nervous system via intravenous injection, they did not differentiate into neural phenotypes. Methods for delivering MSCs for MS-ON currently include intravitreal and systemic injection and intranasal delivery.

2. MSCs and optic neuritis:

(i) Intravitreal administration: An intravitreal injection of MSCs is feasible as a means to reach the dysfunctional retinal ganglion cells. Injection of MSCs into the vitreous in a rodent model rescued the retina from ischemic damage by suppression of apoptosis, preserved autophagy, and attenuation of inflammation and vascular permeability [59–61]. Intravitreally transplanted MSCs may also function by donating functional mitochondria to retinal ganglion cells [62]. Similarly, neural stem cell-based factors, glial derived neurotrophic factor (GNTF) or ciliary neurotrophic factor (CNTF), synergistically protected injured RGCs from cell death in an optic nerve crush model [63]. The main drawback of this approach is that the MSCs did not enter the optic nerve [64].

(ii) Parenteral administration: A recent animal study in mice with EAE showed promising results with MSCs for treatment of ON. Mice received 10^6^ cells intraperitoneally. There was significantly lower motor-sensory impairment, improved pattern electroretinogram, and preserved retinal nerve fiber layer. Increased expression of Abca1, a cholesterol efflux regulatory protein, and reduced HIF-1 were present, although the functional roles of these gene expression alterations were not examined, leaving the mechanism of improvement still uncertain [65].

In a Phase IIa open label study, intravenous administration of autologous MSCs (mean dose of 1.6 × 10^6^ cells/kg) improved visual function in MS patients with optic neuritis. Visual acuity, visual evoked response latency and optic nerve area improved, and retinal nerve fiber layer was preserved. This study lacked a control group and could not differentiate between treatment and the natural course of the disease [66]. In an observational study of 15 patients with NMOSD, 10^8^ autologous MSCs were given intravenously, and T2 or gadolinium-enhancing T1 lesions decreased in the optic nerve. Visual acuity, retinal nerve fiber layer thickness, and optic nerve diameter increased [67].

(iii) Intranasal administration: Intranasal administration of stem cells and other drugs bypasses the blood brain barrier, enabling non-invasive delivery to the optic nerve and brain [68]. While there are not yet any studies of intranasal MSCs in ON, there are promising results from pre-clinical studies in other neurodegenerative diseases. Bone-marrow MSCs preconditioned with fasudil suppressed immune response in a mouse model of Parkinson’s disease, decreasing dopaminergic neuron loss and improving motor function. MSCs migrated to hippocampus and substantia nigra for up to 1 month after intranasal administration [69]. Improved migratory ability of MSCs following intranasal administration was also noted in a study of cerebral ischemia in mice. The co-delivery of MSCs and insulin growth factor (IGF-1) resulted increased neurogenesis and angiogenesis in the ischemic region, leading to improved functional recovery [70]. Other examples of intranasal MSC administration include in models of neonatal stroke, encephalopathy of prematurity, Alzheimer’s disease and EAE [71–74].

Validating this approach for ON is that intranasal administration of MSC secretome to mice with EAE-induced ON attenuated inflammation, demyelination, and RGC loss [75–77]. ST266, given intranasally to EAE mice resulted in decreased vision loss, inflammation, and demyelination. ST266 is derived from amnion-derived multipotent progenitor cells, a subpopulation of amnion epithelial cells grown in serum free media. The concentration of proteins in ST266 ranges from the pg/mL to ng/mL range, and total concentration of secretome proteins is about 100 μg/mL [78]. Filtered ST266 with elements < 50kD did not have significant effects in the retina; the effects could be due, at least in part, to the presence of EVs in the mixture (see below). There was localized accumulation of ST266 in the retina and optic nerve, and significant neuroprotection of RGCs [79]. Intranasal ST266 in mice with optic nerve crush rescued RGCs, decreased demyelination, and provided neuroprotection for up to 10 days [80]. The exact routes of transport of drugs after intranasal administration still remain under investigation [81]. ST266 is currently in Phase I trials via intranasal administration in patients with intraocular hypertension who have not yet developed optic nerve damage [49].

Use of MSCs is limited by their cellular senescence [82], possibility of malignant transformation in long-term culture [83], and low survival rate in tissues [84]. Most mesenchymal stem effects are paracrine, likely mediated by extracellular vesicles (EVs) [85–87]. Administration of MSCs or their secretome, which demonstrate neuroprotective properties [88], suggests EVs can serve an alternative cell-free strategy for restoring axons and their myelination [72].

### Mesenchymal stem cell-derived extracellular vesicles (EVs)

EVs secreted by living cells mainly include exosomes (50–150 nm diameter) and microvesicles (100–1000 nm) [89]. MSCs are amongst the largest cellular producers of EVs [90]. Although originally believed to be mediators of cellular homeostasis by secreting cellular waste [91], recent studies have highlighted important roles of EVs in intracellular communication and as modulators of cellular immunity, cancer biology, and regeneration [91, 92]. The EV membrane’s lipid bilayer is a subset of the plasma membrane [93]. EVs contain messenger RNA (mRNA), microRNA (miRNA), cytosolic, and trans-membrane proteins [94]. Exosomes are the smallest subset of EVs and play a significant role in the transfer of biomolecules such as RNA, proteins, enzymes, and lipids in physiological and pathological conditions [95, 96].

A defining characteristic of exosomes is their endosomal origin [97]. Exosome production starts with reverse membrane invagination and processing in multivesicular bodies (MVBs), followed by release into the intercellular fluid when MVBs fuse with the cell membrane. All exosomes share a common set of proteins, tetraspanins (CD9, CD63, CD81), Alix, and TSG101, but also contain cargo reflective of the parent cell [96, 98]. When endocytosed by effector cells, EVs trigger cellular responses reflective of their cell of origin [99, 100].

As a cell-free therapy, EVs have potentially significantly greater safety and specificity compared to stem cells [101], can be safely administered cross-species without immunosuppression [102–104], and are efficient small molecule carriers that can deliver anti-inflammatory agents [105–107]. EVs can pass through the blood brain barrier [105], making them suitable for CNS treatment. MSC-EV stability, biocompatibility, and low toxicity make them a favorable direction for research in drug delivery and precision medicine. However, challenges exist in large-scale exosome production, isolation, and storage stability [108]. Below we will cover recent findings on the neuroprotective role of MSC-derived EVs and discuss the plausibility of MSC-EVs as a translationally relevant cell-free therapy for MS- and related disease induced ON.

### Therapeutic effects of EVs in neurodegenerative diseases

Neuronal EVs, including those secreted by oligodendrocytes, are involved in signaling between neural circuits to promote development and function by increasing neurogenesis, synaptogenesis, and network activity [109]. Microglia-derived EVs promote production of ceramide and sphingosine to enhance excitatory neurotransmission, which supports physiological modulation of synaptic activity by microglia [110, 111]. Astroglial (astrocyte) EVs play key roles in regulating extracellular glutamate levels and modulating synaptic activation. MSC-derived EVs are involved in a wide variety of physiological processes including inhibiting natural killer cells, B cells, and mitogen activated T cells [112, 113], moderating microglia and macrophage polarization [114, 115], and reducing oxidative stress [116]. They also function in tissue regeneration and myelin membrane biogenesis [117–121]. While EVs derived from various types of neuronal cells have been studied as possible therapy for CNS diseases, they are applied as drug carriers or must be genetically modified to induce desired effects in target cells [106, 122]. In contrast, MSC-EVs’ inherent roles in immune modulation, neuroprotection, and anti-inflammatory mechanisms make them an ideal choice for the treatment of ON and other neurodegenerative disorders. The relative efficacy of these EVs derived from different cell types remains a significant knowledge gap in the field.

Intranasally administered EVs have shown promising results in rodent models of Alzheimer Disease, and other models of neurodegeneration [123–126]. Other studies found intranasal administration of EVs reduced neuroinflammation and neurodegeneration in models of MS [106], Parkinson’s Disease [127], and prevented cognitive dysfunction in a model of status epilepticus [128]. Bone marrow human MSC-derived exosomes were given intranasally to 5XFAD transgenic mice in a model of Alzheimer Disease, and saw decreased amounts of Aβ plaques and increased memory. To localize the exosomes in the brain, the investigators stained used a fluorescent label of the exosome membrane. Immunohistochemistry showed high fluorescence in the pre-frontal cortex of treated mice 24 h post-treatment, demonstrating the ability of exosomes to reach the CNS via intranasal administration [123]. EVs have been administered into the vitreous to access the retina, but no studies have been performed yet with intranasal administration directly targeting the optic nerve [104, 129, 130]. Novel methods of modifying surface receptors and contents of EVs to better target and protect the retina and optic nerve are currently being developed, which will be discussed below, and present a promising outlook for the application of EVs for the treatment of ON.

### Mechanisms of MSC-EV activity relevant to ON

1. Anti-inflammation

Increasing evidence suggests that EVs attenuate neuro-inflammation through regulating T-cells, macrophages, astrocytes, and microglia [131–137]. Studies on MSC-EVs in MS and neurodegenerative disease models are summarized in Table 1. MSC-EVs improved functional outcome and decreased levels of neuroinflammation and demyelination by reducing pro-inflammatory cytokines as well as CD4 + T cells infiltrating the spinal cord in EAE mice [132, 138]. EVs suppressed T-cell proliferation and induction of regulatory T cells (Tregs), whose deletion or mutation play an important role in the pathogenesis of EAE [138, 139].

**Table 1:** MSC-EVs as potential therapy for MS, and related neurodegenerative diseases

Disease	Model	Administration	Molecular mechanism	Effect	References
MS	EAE Rat	Intravenous	Regulated polarization of microglia M1-M2	Attenuated demyelination and decreased inflammation	[141]
	EAE Mice	Intravenous	Reduced T-cell proliferation and increased regulatory T-cell activity through anti-inflammatory cytokines	Improved functional outcomes and clinical score, reduced demyelination	[138]
	EAE Mice	Intravenous	Induced anti-inflammatory TH-2 cytokines and TGF-β	Modulated immune response and induced peripheral tolerance	[132]
	TMEV-IDD Mice	Intravenous	Decreased Th-1/Th-17 cytokines	Improved motor deficits	[131]
Alzheimer	APP/PS1 Mice	Intravenous	Inhibited astrocyte activation, decreased pro-inflammatory and increased anti-inflammatory cytokines	Alleviated plaque deposition and Aβ accumulation, improving cognitive function	[148]
	3xTg Mice	Intranasal	Regulated polarization of microglia M1-M2	Neuroprotective effects, increased dendritic spine density	[143]
Status Epilepticus	Pilocarpine Mice	Intranasal	Decreased glutamatergic and GABAergic neurons, regulation of pro/anti-inflammatory cytokines	Neuroprotective effects, preserved cognitive and memory function	[128]
Stroke	MCAO Rats	Intravenous	miR-133b regulated CTGF in astrocytes, increasing axonal plasticity	Induced functional recovery	[149]
Glaucoma	ONC Mice	Intravitreal	Improved retinal ganglion cell survival, downregulated cis-p tau	Improved cognitive visual behavior	[150]
	ONC Rats	Intravitreal	Modulated inflammatory response through miR-based mechanisms	Neuroprotection of retinal ganglion cells	[130]

CTGF, Connective tissue growth factor; EAE, Experimental animal encephalomyelitis; MCAO, Middle cerebral artery occlusion; MS, Multiple sclerosis; ONC, Optic nerve crush; TMEV-IDD, Theiler’s murine encephalomyelitis virus induced demyelinating disease

As the resident immune cells of the CNS, microglia play an important role in initiating and attenuating neuroinflammation. Mounting evidence suggests the role of MSC-EVs in mediating immunomodulatory effects by regulating microglial activity and promoting a shift in microglial phenotypes from M1 (pro-inflammatory) to M2 (anti-inflammatory) [131, 136, 140]. For example, MSC-EVs administered intravenously to EAE mice improved symptoms, attenuated demyelination, and decreased neuroinflammation by regulating polarization of microglia between M1 and M2 phenotypes [141]. In a murine model of Alzheimer’s disease, MSC-EVs inhibited the activation of microglia through transfer of microRNAs-467f and -466q, decreasing expression of pro-inflammatory cytokines [142]. Intranasal administration of MSC-EVs to a 3xTg mice model of Alzheimer’s disease reduced pro-inflammatory cytokine secretion from microglia, while enhancing the secretion of anti-inflammatory cytokine IL‐10 [143].

2. Axon remyelination and MSC-EVs

In MS and related disorders, activated immune cells in inflammatory lesions damage myelin-producing oligodendrocytes, causing demyelination. Several studies have shown MSC-EVs’ ability to protect and promote the development of OLGs and their precursor cells throughout the course of the disease. EAE mice injected with MSC-EVs had improved motor function and decreased demyelination in the spinal cord. EVs also promoted differentiation of OLG precursor cells into mature OLGs [144]. But this study has significant limitations. There was large variability in onset of EAE, with significant sex differences in the onset and response to treatment. The neuroprotective effects of MSC-EVs on oligodendrocytes were also reported in models of ischemic brain injury. MSC-EVs boosted remyelination and increased mature oligodendrocyte and neuronal cell counts, leading to significantly improved learning ability in mice with perinatal brain injuries [10]. Mice exposed to hypoxia were injected with EVs which attenuated demyelination, increased proliferation of endothelial cells, and increased the number of mature OLGs [145]. The neuroprotective effects of the EVs were largely attributed to their ability to modulate activation of microglia and astrocytes. In TMEV-IDD (TMEV-induced demyelinating disease) mice, MSC-EVs reduced brain atrophy, and cytokine levels [131].

It is important to note that most of these studies are limited by inability to track the effects of EVs over an extended period, and more importantly, that EVs typically do not last long in tissues [10, 103, 131, 141–143]. Thus, the temporal sequence of improvement is difficult to assess. Fluorescent tracking of EVs after intravenous injection found that the administered EVs collected largely in the spleen and liver in as little as two hours, and no significant amounts remained in the CNS after a week [131, 138].

Analysis of cytokine levels from the in vivo studies is also limited, as mRNA expression was analyzed in total tissue lysates, not allowing conclusions about their cellular source [138, 143, 145, 146]. More research on uptake and distribution of EVs in disease models will allow better understanding of their full potential, limitations and explain discrepancies between in vivo and in vitro results [139, 142].

There also is a need to develop EVs formulations that possess extended periods of uptake into tissues. Initial studies by our group in bone regeneration using EVs tethered to hydrogels have shown the ability to produce an extended-release formulation [147]. More discussion of the means to prolong delivery of EVs appears below.

3. MicroRNA mediated EV functionality

The pathology of MS is influenced by histone modifications and gene regulation by microRNAs (miRNA) [151]. Thus, microRNAs are emerging as important mediators in MS and related disorders (Table 2) [152, 153]. Although exosomes carry proteins, mRNAs, and other non-coding RNAs, microRNAs are among the most important components of exosomes that mediate changes in cellular signaling. Exosomes can play a role as carriers of miRNAs to therapeutically regulate MS pathology. Additionally, overexpression of proteins that modulate exosomal miRNA gene expression profiles have the potential to improve therapeutic effects of exosomes [108].

**Table 2:** MicroRNAs in MS and demyelination animal models

miRNA	Disease Model	Effect in Diseased Model	References
miR-21	Pediatric Multiple Sclerosis	Increased levels in white matter	[154]
miR-219	Demyelinating model	CNS myelination and remyelination after injury, overexpression promotes early oligodendrocyte maturation	[155]
miR-219	Multiple Sclerosis	Necessary for production of myelinating oligodendrocytes	[156]
miR-146	Multiple Sclerosis (EAE Model)	Crossed the Blood Brain Barrier and significantly improved functional recovery	[157]
miR-197	Pediatric Multiple Sclerosis	Essential for maintaining T-cell count in patients treated with IFN-beta	[154]
miR-200c	Pediatric Multiple Sclerosis	Increased levels in white matter	[154]
miR-326a	Multiple Sclerosis	Increased levels in T-cell derived EVs in MS patients	[158]

MicroRNAs mediate post-transcriptional gene silencing and are involved in cellular activities including proliferation, differentiation, and migration, as well as disease initiation and disease progression. MicroRNA appears to mediate most EV effects [159] by three known mechanisms: [1] Binding the 3’UTR of their target mRNA, silencing the gene and blocking translation, [2] Translational repression by cleaving and degrading mRNA, [3] De-adenylation and degradation of mRNAs [160]. The miRNA processing endonuclease Dicer is a member of the ribonuclease III family that functions in the RNA interference pathway to cleave long double stranded RNA molecules into small RNAs including miRNA and siRNA [161, 162]. Dicer1 is essential to the miRNA pathway and Dicer2 facilitates the siRNA pathway [161]. Dicer cleaves the precursor miRNA (pre-miRNA) hairpins at the stem-loop boundary, generating mature miRNA [162]. ADicer knockout mice (ADicerKO) lack effective miRNA processing in adipose tissue. EVs from ADicerKO mice exhibited significant alterations in 422 EV miRNAs [163].

MicroRNA-219 promoted oligodendrocyte maturation and regeneration in the EAE model [155]. Its gene targets were oligodendrocyte inhibitors including Nfia, Nfib, and Lingo1 in OPCs. Similar findings have been found in an animal model of Krabbe’s disease [164]. MicroRNA-125a-3p was upregulated in MS patients and in OPCs from the spinal cord of EAE mice; blocking this microRNA accelerated remyelination [165]. Similarly, increased levels of miR-27a were found in OPCs from MS patients and in animal models of demyelination [166]. Studies on microRNAs in MS and related disorders are summarized in Table 2. MicroRNA regulation of OPCs is an emerging field with significant implications for design of treatment strategies in MS. In addition to viral transfection [165], another means to deliver microRNAs is via EVs.

### Modification of EVs for targeted delivery and functionality

Regardless of the route of administration, EVs are highly promiscuous and tend to enter the first cells they encounter. This can limit the ability of EVs to reach cells that are further from the site of administration. The surface of EVs can be modified to carry ligands to take advantage of the presence of specific receptors on the cell surface for targeting and thereby deliver the EVs to the desired cell types. The first use of this methodology was engineering EVs from dendritic cells to express neuron-specific rabies viral glycoprotein peptide, to bind the acetylcholine receptor on neurons [167]. EVs conjugated to a monoclonal antibody against GAP43 targeted delivery of quercetin to ischemic neurons [168]. Another example was T7, a transferrin receptor-binding peptide attached to the EV surface, which resulted in targeting of gliomas [169]. With respect to MS specifically, carboxylic acid-functionalized LJM-3064 aptamer was covalently conjugated to amine groups on the EV surface, which resulted in proliferation of OLGs in vitro. In mice, these EVs suppressed inflammatory response and decreased demyelination [170]. Targeting of EVs, while an exciting technology, still faces significant technical hurdles. Chemical modifications of surface proteins and addition of monoclonal antibodies can alter endocytosis of EVs [171, 172].

An alternate approach is genetic modification, where the EV surface is modified using a lentiviral carried plasmid for transfection of the parent cells, as we and others previously reported [173–175]. With the genetic modification, a stable cell line of, e.g., MSCs, can be created, from which EVs of predictable composition and properties can easily be obtained [147].

Functionally engineered EVs (FEEs), where EVs are modified to carry proteins or specific miRNAs, are also emerging as a potential nano-therapeutic tool in the brain. In several different models, EVs overexpressing the miR17-92 cluster enhanced function and myelination [176, 177]. There are few studies available on the efficacy of functionally engineered MSC-EVs in EAE. Experiments have involved EAE mice with engineered EVs derived from phagocytes. In Zhuang et al.’s study, macrophage-derived EVs were complexed with the anti-inflammatory drug curcumin, and administered intranasally to EAE mice [106]. The EV-curcumin complex significantly reduced neural inflammation and disease severity score by lowering the number of activated inflammatory microglial cells (CD45.2 + IL-1β +). Engineered BV-2 microglia released EVs containing IL-4, an anti-inflammatory cytokine, which targeted phagocytes through the overexpression of Mfg-8 on its surface [122]. A single injection of the EVs in EAE mice reduced neuroinflammation, improved clinical score, and attenuated tissue damage by up-regulating anti-inflammatory markers. Engineered extracellular vesicles from HEK 293 T cells overexpressing miR-219a-5p increased oligodendrocyte precursor cell differentiation and severity of EAE [178].

### Prolonging EV delivery

With a short half-life in tissues, one of the biggest challenges in developing EVs for therapeutics is to enhance their residence in the tissues. Loading of EVs into hydrogels for sustained release has been attempted in several organ systems. Hydrogels increase the efficacy of EVs by concentrating them in a single area. Li et al. encapsulated miR-26a-enriched MSC EVs in a biodegradable hydrogel made of thiol-modified hyaluronan (HA), heparin (HP), gelatin, and polyethylene glycol (PEG) and implanted them into calvarial defects in mice [179]. The hydrogel complex provided a controlled release of the miR-enhanced EVs, successfully integrated with the host bone marrow cells, and sustained an upregulation of miR-26a expression, leading to greater vascularization, bone regeneration, and complete repair of the calvarial bone defect. Other research groups have delivered EVs in a number of different biomaterials to target various disease models (Table 3). With the conjugated systems, sustained release of EVs was maintained for 14–36 days [180, 181]. The longer period of time and improved integration of EVs lead to significantly higher levels of wound healing, tissue repair, and blood perfusion, as well as decreased inflammation and apoptosis.

**Table 3:** EV-hydrogel delivery systems

Type of EV	Hydrogel Material	Application	References
BMSC-EV	Thiol-modified hyaluronan, heparin, gelatin, and polyethylene glycol	Increased levels in white matter	[179]
HMSC-EVs	Photoinduced imine crosslinking hydrogel	Cartilage regeneration and repair	[180]
ADSC-EVs	Pluronic F127, oxidative hyaluronic acid, and Poly-ε-L-lysine	Chronic diabetic wound healing	[181]
UMSC-EVs	Silk fibroin hydrogel	Aging-induced vascular dysfunction	[182]
PMSC-EVs	Chitosan hydrogel	Hindlimb Ischemia	[133]
GMSC-EVs	Chitosan and silk-based hydrogel	Diabetic wound healing	[183]
UMSC-EVs	PA-GHRPS and NapFF peptide hydrogel	Myocardial infarction	[182]

ADSC: Adipose tissue-derived mesenchymal stem cells; GMSC: Gingival mesenchymal stem cells; PMSC: Placental mesenchymal stem cells; UMSC: Umbilical cord-derived mesenchymal stem cells

Natural and synthetic hydrogels allow for precise control of the types of materials, its shape, and the timing and level of its degradability. Coupled with its ability to enhance the therapeutic effects of exosomes, hydrogel-EV systems have strong potential for clinical application.

### MSC-EVs: comparison to MSCs and challenges for clinical translation

Compared to the MSCs from which they are derived, EVs have shorter lifespan and thus need for multiple injections [103, 130]. MSCs can be injected or placed at the site of action, and while they may not integrate into the tissue, they may remain intact for a longer period of time. For example, after injection of CD + 34 MSCs into the vitreous, the cells were found to have migrated into the retinal vasculature after about 4 months [184]. However, immunosuppression is needed to maintain the survival of the stem cells [185].

MSCs also have a migratory capacity [186], while for EVs studies of migration within tissues still remain incomplete. We found that MSC-EVs injected into the vitreous did not penetrate any deeper than the outer plexiform layer. (This is not, however, a disadvantage for access to inner retina and retinal ganglion cells) [103]. MSCs and EVs have different considerations for long term storage. Storage at low temperature (− 80 °C) may affect the stability of EVs [187]. Lyophilization or use of cryoprotective agents may mitigate these problems. With MSCs, cryopreservation may also impair their function [188]. Recent reviews have discussed these issues and novel means to preserve MSC function [189].

A major challenge with EVs is the accuracy of the methods in measuring the quantity and purity of exosomes. The different methods for quantitation of amount of EVs and their purity, as well as preparation methods, have been reviewed thoroughly elsewhere [190]. For regulatory approval, assuring purified preparations is essential. EV production requires culturing the parent cell line, harvesting EVs from conditioned media, and separation/purification of contaminants. Large scale consistent production necessitates use of a bioreactor, to facilitate cell cultivation and continuous medium collection [191]. Good manufacturing practice will be essential for large scale production of EVs [192].

## Conclusions

Demyelinating optic neuritis is a classic presentation of multiple sclerosis and related conditions including neuromyelitis optica spectrum disorder and anti-myelin oligodendrocyte glycoprotein associated disease. ON pathology is marked by an abnormal optic nerve and inflammatory demyelination. Clinical research has revealed efficacy of immunosuppressive treatment and monoclonal antibodies targeting different inflammatory pathways. However, achieving neuroprotection of glial cells and RGCs and remyelination are needed. Mesenchymal stem cell therapy is a promising approach that results in anti-inflammatory effects and remyelination and has shown success in some small clinical studies. The main limitation of MSC therapy for ON is the difficulty of reaching the site of pathology in the optic nerve and retina. This necessitates investigation of a cell-free approach that utilizes the paracrine effects of EVs produced by MSCs, which have the ability to cross the blood–brain barrier. EVs have potential to be an effective cell-free therapy because of their increased specificity and ability to be safely administered without immunosuppression. EV cellular uptake can be enhanced through genetic engineering of their parental cells. MSC-EVs decreased inflammation and enhanced remyelination in animal models of MS. Further investigation has found the significant role played by microRNA in the mediation and progression of MS. Overexpressing specific miRs promoted remyelination. Delivery of engineered EVs can be prolonged through a robust hydrogel-EV system, which sustains delivery of EVs to the target site. Further exploration of the potential for EVs enhanced through miRNA modification, functional engineering, and engineered hydrogel-EV systems is necessary to develop effective clinical translation for MS and related diseases related ON.

## Data Availability

Not applicable.

## Abbreviations

aHSCT: Autologous hematopoietic stem cell transplant
2ndAD: Secondary autoimmune diseases
BBB: Blood brain barrier
CNS: Central nervous system
CNTF: Ciliary neurotrophic factor
CSF: Cerebrospinal fluid
C1q: Complement component 1q
EAE: Experimental autoimmune encephalomyelitis
EVs: Extracellular vesicles
GNTF: Glial derived neurotrophic factor
HA: Haluronan
HP: Heparin
iNOS: Inducible nitric oxide synthase
IGF: Insulin growth factor
IL: Interleukin-1α,
ISEV: International Society for EVs
TNF: Tumor necrosis factor-α
MCAO: Middle cerebral artery occlusion
MSC: Mesenchymal stem cell
mRNA: Messenger RNA
miRNA: MicroRNA
MR: Magnetic resonance
MOGAD: Anti-myelin oligodendrocyte glycoprotein associated disease
MS: Multiple sclerosis
MVBs: Multivesicular bodies
NK: Natural killer cells
NMO: Neuromyelitis optica
NMOSD: Neuromyelitis optica spectrum disorder
OLGs: Oligodendrocytes
OCT: Optical coherence tomography
ONC: Optic nerve crush
OPCs: Oligodendrocyte precursor cells
PEG: Polyethylene glycol
RGC: Retinal ganglion cell
RNFL: Retinal nerve fiber layer
siRNA: Small interfering RNA
Tregs: Regulatory T cells
TMEV-IDD: TMEV-induced demyelinating disease
VEP: Visual evoked potential
